# Physician requests by patients with malignant pleural mesothelioma in Japan

**DOI:** 10.1186/s12885-019-5591-7

**Published:** 2019-04-25

**Authors:** Yasuko Nagamatsu, Isao Oze, Keisuke Aoe, Katsuyuki Hotta, Katsuya Kato, Junko Nakagawa, Keiko Hara, Takumi Kishimoto, Nobukazu Fujimoto

**Affiliations:** 1 St. Luke’s International University, Graduate School of Nursing Science, 10-1 Akashicho, Chuo-ku, Tokyo, 1040044 Japan; 2 Division of Molecular and Clinical Epidemiology, Aichi Cancer Center Research Institute, 1-1 Kanokoden, Chigusa-ku, Nagoya, 4648681 Japan; 3 Department of Medical Oncology, National Hospital Organization Yamaguchi-Ube Medical Center, 685 Higashikiwa, Ube, 7550241 Japan; 4 Center for Innovative Clinical Medicine, Okayama University Hospital, 2-5-1 Shikatacho, Okayama, 7008558 Japan; 5 Department of Radiology, Kawasaki General Medical Center, 2-6-1 Nakasange, Okayama, 7008505 Japan; 6 Department of Nursing, Okayama Rosai Hospital, 1-10-25 Chikkomidorimachi, Okayama, 7028055 Japan; 7 Department of Medicine, Okayama Rosai Hospital, 1-10-25 Chikkomidorimachi, Okayama, 7028055 Japan; 8 Department of Medical Oncology, Okayama Rosai Hospital, 1-10-25 Chikkomidorimachi, Okayama, 7028055 Japan

**Keywords:** Asbestos, Communication, Mesothelioma, Patient-centered care, Support

## Abstract

**Background:**

Malignant pleural mesothelioma (MPM) is a fatal and rare disease that is caused by the inhalation of asbestos. Treatment and care requests made by MPM patients to their physicians were collected and analyzed.

**Methods:**

This cross-sectional survey was part of a larger study (*N* = 133) regarding the quality of life of MPM patients. Specific responses to two open-ended questions related to patients’ requests regarding treatment and care were quantified, analyzed and divided into categories based on content.

**Results:**

Responses (*N* = 217) from MPM patients (*N* = 73) were categorized into 24 subcategories and then abstracted into 6 categories. The majority of requests were related to patient-physician communication. Patients wanted clear and understandable explanations about MPM and wanted their physician to deliver treatment based on the patient’s perspective by accepting and empathizing with their anxiety and pain. Patients expected physicians to be dedicated to their care and establish an improved medical support system for MPM patients.

**Conclusion:**

Patients with MPM had a variety of unmet needs from their physicians. Physicians who provide care to MPM patients should receive training in both communication skills and stress management. A multidisciplinary care system that includes respiratory and palliative care for MPM patients should be established.

**Electronic supplementary material:**

The online version of this article (10.1186/s12885-019-5591-7) contains supplementary material, which is available to authorized users.

## Background

Globally, exposure to asbestos in the workplace is now considered one of the main causes of work-related deaths with one-half of these deaths attributable to cancers, including malignant pleural mesothelioma (MPM) [1]. The number of deaths from MPM in Japan was greater than 1400 in 2015 [2]. This number is expected to grow by 2040 [3]. MPM is fatal [4, 5] and causes debilitating physical symptoms, such as pain, dyspnea, fatigue, loss of appetite, and sweating [6]. Patients with MPM also experience emotional difficulties, including the shock of diagnosis [7], anxiety and depression [8], or guilt and shame [9]. In addition, patients have complained of a lack of information about the disease and a lack of compensation from their insurance providers [10]. Patients have also expressed anger toward their employers who did not alert them to the hazards of asbestos [8, 11], in response to their own ambivalence toward working in an unhealthy environment versus supporting their family [8], and as a result of the stress of dealing with asbestos-related lawsuits [8, 12, 13]. For patients with MPM, a multidisciplinary approach involving a psychologist specialized in taking care of cancer patients and their families is recommended [14]. In Japan, physicians are the major source of information and support for patients with MPM. Unfortunately, some patients with MPM have not been well informed, and physicians were unable to meet their needs. This lack of rapport and communication eventually led to dissatisfaction with their attending physician and had a negative impact on patients’ quality of life (QOL) [10]. Given the importance of the physician-patient relationship, it is important to further investigate what MPM patients need from their physicians to address their current gap in knowledge of the disease. The current study is part of a larger study regarding the QOL of patients with MPM. The aim is to determine the needs of patients within the health services by quantifying the requests to their physicians and qualitatively analyzing their answers to two open-ended questions regarding these requests.

## Methods

### Study design

This study is a part of a major study about QOL and intention of care among MPM patients. This study is a cross-sectional descriptive study that used a mailed survey [15]. In brief, an invitation to participate in the study was sent to 422 cancer hospitals in Japan; 64 hospitals (15.2%) agreed to participate. In February 2016, the participating hospitals distributed 438 questionnaires to their patients with MPM. Additional questionnaires were mailed in March 2016 to 94 MPM patients who were identified through patient and family support groups, which have 15 branches in Japan. The completed questionnaires were mailed back to the researchers by the end of April 2016. Basic demographic and medical data of the participants were gathered using a separate researcher-constructed, patient self-administered questionnaire. The questionnaire contained 72 questions regarding the QOL of MPM patients and related factors. In total, 88 (20.1%) questionnaires were returned. Of the 94 questionnaires that were sent to the patients and family support groups, 45 (47.9%) were returned. In total, 133 questionnaires were collected, and 73 (54.9%) participants answered the two open-ended questions referred to as “requests to physicians.” Table 1 describes the characteristics of the participants. In the current study, we evaluated the answers to open-ended questions: (1) “What do you request from your doctor about your diagnosis and treatment?” and (2) “Describe the attitude and words you want from your doctor (Additional file 1).”

**Table 1:** Demographic and Medical Characteristics of the Study Participants (*N* = 73)

Characteristic	Response	n	%
Gender	Male	61	83.6
	Female	12	16.4
Age in years (mean ± SD)		66.8 ± 11.3	
MPM Treatment Received			
Surgery	I did not have	43	58.9
	I had	30	41.1
Chemotherapy	I never had	13	17.8
	I had before	29	39.7
	I am having now	31	42.5
Radiotherapy	I never had	52	71.2
	I had before	19	26.0
	I am having now	2	2.7
Palliative care	I never had	39	53.4
	I had before	9	12.3
	I am having now	25	34.2
ECOG Performance Status	0	12	16.4
	1	40	54.8
	2	7	9.6
	3	13	17.8
	4	1	1.4
Relationship with Their Physician	Very good	30	41.1
	Good	31	42.5
	Moderate	9	12.3
	Not very good	2	2.7
	Poor	1	1.4

ECOG, Eastern Cooperative Oncology Group; SD, standard deviation

### Data analysis

Basic medical and demographic information was tallied, and the percentages and mean values were calculated. The answers to the questions were analyzed using the qualitative content analysis procedures of Graneheim and Lundman [16]. Initial categories were created by grouping similar words and phrases. The authors discussed the definitions and examples that emerged through the content analysis to enhance the representation and add clarity to categories, definitions, and examples. Responses that were not easily ascribed to a specific category were discussed and assigned to an appropriate category when the research team achieved 100% consensus. This process was repeated until all the responses were coded [17]. Finally, two researchers verified all the answers and tallied the number of times each category and subcategory was mentioned. The prevalence was compared between patients who received palliative care and those who did not receive palliative care. Comparisons between independent groups were performed using the chi-square test.

### Ethical considerations

Ethical approval for the study was obtained from the Okayama Rosai Hospital Ethics Review Board. Eligible MPM patients received written information about the study, including their right to confidentiality, to refuse participation, or to withdraw at any point in the study without penalty.

## Results

### Requests to the physician

The 217 requests by 73 respondents were categorized into 24 subcategories and were finally integrated into six categories. Table 2 displays the categorized requests to physicians by MPM patients.

**Table 2:** Requests to Physicians by MPM Patients (217 requests; *N* = 73)

Categories		Times mentioned	% of Sample
Subcategories			
1. Understandable explanation to meet the patient’s needs		80	
1.1	Explain the cause of the symptoms, curability and prognosis of the disease, and provide a treatment plan	41	56.2
1.2	Use simple words	12	16.4
1.3	Explain the purpose, benefits, risk and results of examinations	10	14.0
1.4	Inform about all treatment options	10	14.0
1.5	Give advice about daily activities	3	4.1
1.6	Spend enough time on explanations	2	2.7
1.7	Confirm patient’s understanding and allow them to ask questions	2	2.7
2. Patient-centered treatment		39	
2.1	Minimize the physical impact of treatment	11	15.1
2.2	Do not give up on the treatment	10	14.0
2.3	Respect patient’s intention	9	12.3
2.4	Careful clinical assessment to not miss clinical signs of progression	9	12.3
3. Improvement of treatment and support systems for MPM		35	
3.1	Develop country-wide specialized care system	16	21.9
3.2	Develop new drugs	10	14.0
3.3	Improve information systems	9	12.3
4. Emotional support		32	
4.1	Be kind and cheerful	11	15.1
4.2	Sympathize with patient’s anxiety	10	14.0
4.3	Have a reliable attitude	6	8.2
4.4	Empathy for victims of asbestos	3	4.1
4.5	Visit patient as often as possible	2	2.7
5. Customize “breaking the bad news”		24	
5.1	Tell everything including bad news	17	23.3
5.2	Do not inform about bad news	5	6.8
5.3	Customize the contents and the way of informing	2	2.7
6. Dedication to the treatment of MPM		7	
6.1	Confront intractable disease	4	5.4
6.2	Learn about MPM	3	4.1

MPM, malignant pleural mesothelioma

#### Understandable explanations to meet patient’s needs

Among the 217 requests, 80 concerned explanations from their doctor. The most frequent requests were to tell the cause of the symptoms, explain the curability and prognosis of the disease, and provide a treatment plan (*n* = 41).

*“A doctor told me ‘You have 2 years to go.’ However, I was so healthy and could not imagine how this could be happening. I was in a panic because I did not know what to do next. Later, another doctor said ‘Live as you lived. When you have pain, I will introduce you to a doctor for pain.’ This explanation gave me back my life.”* (#18 Male)

The second most frequent request was to provide information about their disease in simple words (*n* = 12).

*“‘There is no change, the same as the last time.’ [He] does not explain anything. How is it the same? Is it good or bad? Why does he think so? If he based his diagnosis upon data, show them to me.”* (#47 Male)

Patients with MPM exhibited great concern regarding examinations. They wanted their physician to explain the purpose, benefits and risks, and results of examinations (*n* = 10).

*“Explain concretely why I need an examination and do not forget to tell me the results, including my data compared with normal levels. Being well-informed and knowing my results eases my anxiety and gives me a sense of control. I feel that I am not that bad yet.”* (#72 Male)

*“I want to know if the chemotherapy worked on my tumor.”* (#10 Male)

In addition, the respondents wanted to know all the treatment options (n = 10).

*“I need to know the latest treatment.”*(#81 Male)

*“Does any treatment work for patients with MPM?”* (#89 Male)

Furthermore, some respondents wanted advice about preparation. (*n* = 3)

“*My doctor let me know the benefits of palliative care and advised me to introduce it at an early stage. It was helpful because I had time to prepare.”* (#72 Male)

Patients with MPM wanted their physician to spend enough time on explanations (*n* = 2).

*“I know doctors are very busy. However, please understand that each patient needs time to understand what you said. Please do give us information so that we can understand one thing and then go further with the explanation. If you only explain things one-by-one, we never understand and get confused.”* (#2 Male)

Finally, patients with MPM wanted their physician to confirm their understanding of the explanation and allow them to ask questions (n = 2).

“*My doctor always asks me ‘Is there anything you want to ask me?’ You will never know how greatly I appreciate him. It is the greatest gift for patients.”* (#45 Male)

#### Patient-centered treatment

Eleven patients requested the minimization of the physical impact of the treatment.

“*I do not want to suffer from heavy treatment. Just relieve my pain and let me stay at home until the last day.”* (#78 Male)

Other respondents wanted their physician to not give up on treatment (*n* = 10).

*“My doctor said I cannot receive chemotherapy any more, but I really want to receive treatment. I hope my doctor never gives up on my treatment … .I feel safe as long as I receive treatment.”* (#75 Male)

Nine respondents commented that their physician should respect patients’ intentions because they were not treated in the way they wanted.

“*My doctor came to me and said, ‘Move to another hospital. The members of the medical conference decided not to treat you here anymore.’ How can they say that? Patients are completely reliant on their doctors; at the very least, treatment must include the patient’s perspective.”* (#120 Male)

*“I hope my doctor not only treats my tumor but also takes care of me. I am not a box with cancer, but a living person.”* (#123 Male)

Another 9 patients with MPM wanted their physician to perform a careful clinical assessment to not miss clinical signs of progression (*n* = 9).

*“I want my doctor to check very carefully to identify progress as soon as possible because MPM has no effective treatment. However, he repeats the same examination in a mechanical way. This makes me uneasy.”* (#99 Male)

#### Need for improvement of treatment and a support system for MPM

Some patients described specific suggestions to improve support systems. The participants wanted the development of country-wide specialized care systems (*n* = 16), development of new drugs (*n* = 10), and improvement of information systems (*n* = 2).

*“Because MPM is a difficult disease, I want to be treated by a specialist. I am disappointed that there is no specialist in my area.”* (#36 Male)

*“Develop a test for early disease detection and develop a medical care service as soon as possible.”* (#12 Male)

*“We need a liaison to consult with about MPM. It is so hard to collect information about the disease and hospitals for individual patients and their family.”* (#113 Male)

#### Emotional support

The participants wanted their physicians to be kind and cheerful (*n* = 11), to sympathize with patients’ anxiety (n = 10), to have a reliable attitude (*n* = 9), and to visit the patient as often as possible (n = 2).

*“No one can cheer me up but the doctor. I want my doctor to say, ‘it is alright.’ I was so happy when he said, ‘Let’s work together’.”*(#8 Male)

*“When I am very anxious, I ask my doctor the same question many times. He says, ‘I explained that before, didn’t I?’ He is angry, and it makes me more anxious. I hope he allows me to ask questions as many times as I want.”* (#102 Male)

*“My doctor pays attention to the computer and does not look at me. I hope he looks me in the eye.”* (#113 Male)

*“My doctor came to me and smiled at me. It was only for a minute, but it worked and made me feel so relieved. I want him to come as often as possible.*” (#45 Male)

Furthermore, patients with MPM wanted to be considered as a victim of the use of asbestos and expected their physician to have empathy with victims of asbestos (*n* = 3).

*“If I were to die from another cancer, I would not suffer like this. I am so resentful that I will die from asbestos; this feeling prevents me from facing my problems. How dare my doctor say ‘patients with MPM are not the only ones who are suffering?’”* (#106 Male)

#### Customize “breaking the bad news”

Some of the participants wanted their physicians to inform them about everything including bad news (*n* = 17). In contrast, some did not want to be informed about bad news (*n* = 5) or requested that doctors customize the content and way of presenting bad news (*n* = 2).

“*I want my doctor to tell me everything, including bad news.”* (#64 Male)

*“I was already shocked to learn that I have MPM; it was cruel to tell me the time I had left.”* (#112 Male)

*“Don’t tell me the bad news. Just let me know something good.”* (#75 Female)

#### Dedication to the treatment of MPM

Patients wanted their physicians to confront the intractable disease (*n* = 4) and to learn more about MPM (*n* = 3).

*“I hope my doctor has enough ambition and passion to battle the difficult disease of MPM.”* (#127 Male)

*“My doctor’s priority is to make money from us. They do not have the spirit to take care of us on our deathbed.”* (#120 Male)

*“Doctors are the only hope for patients. I beg them to learn more about MPM.”* (#65 Male)

We compared these categorized requests according to MPM patients with or without palliative care. MPM patients who did not receive palliative care described more requests concerning understandable explanations, need for improvement of treatment and support systems, and dedication to the treatment of MPM than those who received palliative care. Among these requests, there was statistical significance concerning communication regarding the cause of the symptoms, curability and prognosis of the disease, and treatment plan (*p = 0.030*) (Additional file 2: Table S1).

## Discussion

This study was part of a larger study about the QOL of MPM patients and sought to reveal their healthcare-related needs, particularly regarding interactions with their physician. Patients with MPM wanted their physicians to provide supportive communication, patient-centered care, and an attitude of dedication and commitment to their treatment. Most requests to their physicians concerned the content and method of communication. Patients wanted precise information about their condition, even if it was raw data from examinations. Patients also wanted the doctor to explain in laymen’s terms how the condition would affect their daily lives. A previous study of patients with MPM also identified the difficulty of physicians in establishing rapport and engaging in a fruitful two-way communication [18]. The style of communication requested by patients with MPM was similar to studies of other cancers: a two-way exchange of information [19, 20]; and communication to provide the patient with data [21, 22]. Additionally, patients wanted to be allowed to ask questions [22], to be treated by physicians with insightful and empathetic attitudes [23, 24], and to be assured of on-going support [24].

The requests for emotional support were clearly evident in this study. The need for physicians to provide emotional support was documented in previous studies [23, 24], including one in which physicians were considered the most important source of psychological support [25]. In particular, our study indicated that MPM patients had an extra need for empathy due to their perception of being victims of asbestos. Additionally, the diagnosis of MPM engendered deep resentment given the circumstances surrounding their exposure to asbestos [10, 12, 26], feelings of injustice [12], and feelings of being traumatized [27].

This study also indicated that many patients with MPM wished for clear and complete information about their disease and its prognosis, while a smaller number of patients wanted the information to be delivered in a more indirect and vague manner. Yanagihara reported that Japanese patients wanted bad news to be minimized and to be conservative [28]. Patients with MPM were reported to have high levels of uncertainty and feelings of a lack of control leading to psychosocial distress since receiving their diagnosis [29]. Physicians should take these differences into account when they present the diagnosis and prognosis of MPM to their patients.

It is fundamental that any treatment is the result of mutual decision-making between the patient and the physician. Our study demonstrated the frustration of some patients with MPM who could not receive chemotherapy due to a safety issue, leaving them feeling not cared for or abandoned. In addition, the current study indicated that patients who did not receive palliative care described more requests than those who received palliative care. One possible explanation would be a difficulty of physicians to tell the curability and prognosis of the disease to the patients. Miyashita et al. evaluated end-of-life cancer care in designated cancer centers and palliative care units and reported that care evaluation score was lower in designated cancer centers than in palliative care units concerning physical care by physician, help with decision making, and knowing what to expect about future condition [30]. Unfortunately, Japan has a limited care system for patients with MPM [31]. An integrated care and support system is urgently needed with a multidisciplinary approach that includes physicians, nurses, psychologists, support groups, and medical social workers.

Patients with MPM also expect their physicians to have updated knowledge about MPM and continued interest in searching for new methods of treatment. Patients certainly did not want their doctor to be stymied or to give up on them. Budych et al. previously indicated that patients with rare diseases prefer that their physician make most of the decisions regarding their care [32].

Limitations of this study include a low participation rate from hospitals (approximately 20%), although approximately half of the questionnaires were returned from the support groups. This study is also biased toward patients in the early stages of MPM and those with a good relationship with their physicians. However, given that other studies support the findings of this research, the risk of this bias is less of a concern. Further research should include a longitudinal, mixed-methods study that utilizes standardized instruments in addition to interviews with patients and physicians to shed more light on the specific needs of both groups.

## Conclusion

This study indicated that patients with MPM had a variety of needs unmet by their physicians, even if they were in the early stages of the disease, and most had good relationships with their physicians. In addition, the current study indicated that patients who did not receive palliative care described more requests than those who received palliative care. Physicians should consider introducing shared decision-making and empathic verbal and nonverbal communication with dedication to the treatment of MPM. Physicians who provide care to MPM patients should receive training in both communication skills and stress management. A multidisciplinary care system that includes respiratory and palliative nurse specialists should be established for patients with MPM.

## Additional file

Additional file 1: Questionnaire about quality of life of people with malignant pleural mesothelioma. (DOCX 17 kb)

Additional file 2: **Table S1.** (DOCX 22 kb)

## Abbreviations

MPM: Malignant pleural mesothelioma
QOL: Quality of life
