# Supplementary material for: Physician requests by patients with malignant pleural mesothelioma in Japan
Source: BMC Cancer. 2019 Apr 25;19:383. doi: 10.1186/s12885-019-5591-7 (PMC6485076; doi:10.1186/s12885-019-5591-7)
Supplement: Supplementary file 1 — Questionnaire about quality of life of people with malignant pleural mesothelioma. (DOCX 17 kb) [file 12885_2019_5591_MOESM1_ESM.docx]

**Questionnaire about Quality of Life of people with Malignant Pleural Mesothelioma**

1. Please answer about yourself.
2. Age: years old
3. Sex: Male Female
4. When were you diagnosed MPM? Year Month
5. Please put circle of the ( ) of the treatment you have received for MPM.
6. Surgery

(　) I did not have

(　) I had

1. Chemotherapy

(　) I never had

(　) I had before

(　) I am having now

1. Radiotherapy

(　) I never had

(　) I had before

(　) I am having now

1. Palliative care

(　) I never had

(　) I had before

(　) I am having now

1. How active are you? Please put circle of the ( ) which is closest to your condition.

( ) Fully active, able to carry on all pre-disease performance without

restriction

( ) Restricted in physically strenuous activity but ambulatory and able to

carry out work of a light or sedentary nature, e.g., light house work, office work

( ) Ambulatory and capable of all selfcare but unable to carry out any work activities. Up and about more than 50% of waking hours

( ) Capable of only limited selfcare, confined to bed or chair more than 50% of waking hours

( ) Completely disabled. Cannot carry on any selfcare. Totally confined to bed or chair

1. How is the relationship with you and your physician?

( ) Very good

( ) Good

( ) Moderate

( ) Not very good

( ) Bad

1. Please write your request to the medical service.
2. Please write your concern, problem or worries regarding your disease and treatment.
3. What is important when you choose the treatment?
4. What do you expect your physician? What attitude or words do you want from your physician?
5. Please write your opinion freely.

Thank you.
