# Supplementary material for: Physician requests by patients with malignant pleural mesothelioma in Japan
Source: BMC Cancer. 2019 Apr 25;19:383. doi: 10.1186/s12885-019-5591-7 (PMC6485076; doi:10.1186/s12885-019-5591-7)
Supplement: Supplementary file 2 — Table S1. (DOCX 22 kb) [file 12885_2019_5591_MOESM2_ESM.docx]

**Supplemental Table 1**

| Categories | | Patients with Palliative care (n=35) | Patients without Palliative care (n=38) | P value |
| --- | --- | --- | --- | --- |
| **1. Understandable explanation to meet the patient’s needs** | |  |  |  |
| 1.1 | Explain the cause of the symptoms, curability and prognosis of the disease, and provide a treatment plan | 15 | 27 | 0.030 |
| 1.2 | Use simple words | 5 | 7 | 0.709 |
| 1.3 | Explain the purpose, benefits, risk and results of examinations | 3 | 7 | 0.258 |
| 1.4 | Inform about all treatment options | 5 | 5 | 0.815 |
| 1.5 | Give advice about preparation | 1 | 2 | 0.639 |
| 1.6 | Spend enough time on explanations | 1 | 1 | 0.922 |
| 1.7 | Confirm patient’s understanding and allow them to ask questions | 1 | 1 | 0.922 |
| **2. Patient-centered treatment** | |  |  |  |
| 2.1 | Minimize the physical impact of treatment | 7 | 4 | 0.218 |
| 2.2 | Do not give up on the treatment | 5 | 5 | 0.815 |
| 2.3 | Respect patient’s intention | 5 | 4 | 0.564 |
| 2.4 | Careful clinical assessment to not miss clinical signs of progression | 5 | 4 | 0.564 |
| **3. Improvement of treatment and support systems for MPM** | |  |  |  |
| 3.1 | Develop country-wide specialized care system | 5 | 11 | 0.164 |
| 3.2 | Develop new drugs | 4 | 6 | 0.654 |
| 3.3 | Improve information systems | 4 | 5 | 0.891 |
| **4. Emotional support** | |  |  |  |
| 4.1 | Be kind and cheerful | 4 | 7 | 0.461 |
| 4.2 | Sympathize with patient’s anxiety | 3 | 7 | 0.258 |
| 4.3 | Have a reliable attitude | 2 | 4 | 0.497 |
| 4.4 | Empathy for victims of asbestos | 0 | 3 | 0.099 |
| 4.5 | Visit patient as often as possible | 0 | 2 | 0.181 |
| **5. Customize “breaking the bad news”** | |  |  |  |
| 5.1 | Tell everything including bad news | 8 | 9 | 0.964 |
| 5.2 | Do not inform about bad news | 3 | 2 | 0.533 |
| 5.3 | Customize the contents and the way of informing | 0 | 2 | 0.181 |
| **6. Dedication to the treatment of MPM** | |  |  |  |
| 6.1 | Confront intractable disease | 3 | 1 | 0.241 |
| 6.2 | Learn about MPM | 3 | 0 | 0.058 |

MPM, malignant pleural mesothelioma
